# Randomized Controlled Trial of Atorvastatin in Acute Influenza in the Emergency Department

**DOI:** 10.5811/westjem.33580

**Published:** 2025-04-29

**Authors:** Maureen Chase, Michael N. Cocchi, Anne V. Grossestreuer, Xiaowen Liu, Jacob Vine, Ari L. Moskowitz, Michael W. Donnino

**Affiliations:** * Beth Israel Deaconess Medical Center, Department of Emergency Medicine, Boston, Massachusetts; † Beth Israel Deaconess Medical Center, Department of Anesthesia Critical Care, Division of Critical Care, Boston, Massachusetts; ‡ Beth Israel Deaconess Medical Center, Department of Medicine, Division of Pulmonary, Critical Care, and Sleep Medicine, Boston, Massachusetts; § Montefiore Medical Center, Division of Critical Care Medicine, New York City, New York

## Abstract

**Objectives:**

We sought to determine whether atorvastatin administration attenuates the inflammatory response and improves clinical outcomes in acute influenza.

**Methods:**

We conducted a randomized double-blind trial administering atorvastatin 40 milligrams or placebo to adults with confirmed influenza for five days between December 2013–May 2018. Patients were primarily enrolled in the emergency department (ED) at an urban, tertiary-care center. Serum was obtained at enrollment and 72 hours for the primary outcome, change in interleukin (IL-6). Patients reported severity of influenza symptoms over 10 days. We used linear mixed-effects models for the primary comparisons.

**Results:**

Of the 116 enrolled patients, 59 received atorvastatin and 57 received placebo. Groups were well-matched including baseline influenza symptom scores and receipt of an antiviral medication. There was no difference between groups in the change in interleukin-6 (IL-6) levels (*P*=0.468). However, there were significant differences in the overall influenza symptom scores, favoring faster resolution in the atorvastatin group (*P*=0.05). For patients presenting within 48 hours of symptom onset, resolution was faster for the overall score (*P* <0.001) and for the fever (*P*=0.001), sore throat (*P*=0.005) and headache (*P*=0.006) components. No safety concerns were identified.

**Conclusion:**

Atorvastatin administration in acute influenza appears safe. We did not find attenuation of IL-6 with atorvastatin. Patients receiving atorvastatin reported improvement in their clinical symptoms at a faster rate than those in the placebo group, particularly in patients presenting within 48 hours of symptom onset. This trial is registered at ClinicalTrials.gov, Identifier: NCT02056340.

## INTRODUCTION

The World Health Organization estimates 3–5 million cases of severe influenza illness worldwide and attributes 250,000–650,000 respiratory deaths to seasonal influenza outbreaks annually. These figures have remained relatively stagnant over time, and incidences of other comorbid conditions spike when influenza is circulating.^1^,^2^ Vaccination represents the best means of preventing outbreaks, but there is no universal influenza vaccine. For infected patients, the remaining treatment options include existing antiviral agents; however, there are several practical challenges with using these agents as a singular line of defense including cost, efficacy late in disease, limited supplies, and emerging resistance.^3^–^6^ Moreover, these current strategies do not directly address the severe inflammatory response elicited by the virus that causes the infection-associated morbidity and mortality.^7^,^8^ Pro-inflammatory mediators such as interleukin 6 (IL-6) are associated with severe disease and death and, therefore, may represent key therapeutic targets in the treatment of acute influenza infection.^9^–^12^

The class of medications referred to as “statins” has a first-line application in reducing cholesterol levels, but they also have pleiotropic anti-inflammatory and immunomodulatory effects. Multiple studies have suggested an association between the use of statin drugs and a reduction in morbidity and mortality in other types of infection.^13^–^16^ While there is less direct evidence for statin therapy in acute influenza infection, the preponderance of population-level, observational studies suggest statins are beneficial.^17^–^24^ We hypothesized that the administration of a statin medication would attenuate the inflammatory response in patients with acute influenza, resulting in 1) decreased levels of circulating pro-inflammatory cytokines and 2) reduction in the severity of illness and time to clinical resolution of influenza symptoms. To test these hypotheses, we performed a randomized clinical trial administering atorvastatin vs placebo to patients with acute influenza infection.

## METHODS

### Study Design

This was a single-center, randomized, double-blind, placebo-controlled trial comparing atorvastatin to placebo in patients with confirmed influenza (ClinicalTrials.gov Identifier: NCT02056340). The study was conducted at Beth Israel Deaconess Medical Center in Boston, Massachusetts, and was approved by our institutional review board. Study participants were randomized to receive either atorvastatin 40 milligrams (mg) or matching placebo capsule orally daily for five days or to a maximum of seven days in those who remained hospitalized.

### Participants

All patients presenting with an influenza-like illness were screened between December 2013–May 2018. Eligible patients were adults ≥18 years with acute influenza confirmed by either a bedside rapid antigen test or a documented hospital laboratory test (direct fluorescence antibody or polymerase chain reaction). Primary screening occurred in the emergency department (ED), but hospitalized patients with a positive influenza test were also screened.

Patients were excluded if they had concomitant or recent (within 30 days) statin medication use; were pregnant; actively breastfeeding; had cirrhosis or acute liver dysfunction with alanine and aspartate aminotransferase (ALT/AST) > 240 international units per liter; had creatinine phosphokinase (CPK) 3x above normal, had an allergy to statin medications; were unable to tolerate oral or nasogastric medications; had a “do not resuscitate” or “comfort measures only” designation; were a member of a protected population; or were otherwise unable to provide written informed consent. Patients were further excluded if they were taking any medications contraindicated with atorvastatin including cyclosporine, HIV protease inhibitors, gemfibrozil, niacin, azole antifungals, clarithromycin, and colchicine. All participants signed a written informed consent.

**Population Health Research Capsule**

What do we already know about this issue?

*Statins have anti-inflammatory properties; observational studies have suggested a clinical benefit for patients taking a statin at the time they contract influenza.*

What was the research question?

*This trial was designed to assess the effect of atorvastatin therapy on inflammatory markers and clinical outcomes in influenza.*

What was the major finding of the study?

*We found no change in the primary outcome measure, interleukin 6. We found a clinical benefit favoring faster resolution of symptoms in the atorvastatin group, particularly early in infection (P <0.001).*

How does this improve population health?

*Given their low cost and safety profile, statins may represent a strong adjuvant treatment option in both acute influenza and other viral infections. Further study is needed.*

### Randomization and Masking

A 1:1 randomization scheme was created by an independent biostatistician. Atorvastatin 40 mg and matching placebo capsules were created by our institutional research pharmacy. Study drug and the randomization scheme were maintained in the research pharmacy pending enrollment notification, at which time study drug was dispensed to the research team in blinded fashion for delivery to patients.

### Procedures

Safety labs (AST, ALT, CPK) were checked prior to randomization as part of the screening process, and blood samples were drawn prior to study drug administration for biomarker profiling. Patients admitted to the hospital had study drug stored in a locked location and dispensed at the same time each day and had safety lab testing performed at 24 and 72 hours, as well as blood samples every 24 hours for biomarker profiles throughout their hospital stay. Patients discharged from the hospital had the remaining doses of study drug dispensed in a prescription bottle and were asked to return to our clinical research center at 72 hours for blood sampling for both safety labs and biomarker profiles. Any patient with an elevation in safety lab testing above the specified parameters was discontinued from the trial.

Patients were also asked to rate their influenza symptoms (fever, cough, sore throat, headache, myalgias) ranked from 0 to 3 (none, mild, moderate, severe) for a daily score ranging from 0–15 for 10 days.^25^ This daily symptom score served as the basis for assessment of clinical outcomes between groups. If patients remained in the hospital, their daily diary scores were collected in person on paper by a trained research assistant. Patients discharged from the hospital were prompted electronically via email (or by telephone for those who did not use email) to both take their study medication and complete their daily symptom diary.

### Outcomes

The primary endpoint was to determine whether statin therapy reduces the inflammatory response to acute influenza infection. To achieve this aim, inflammatory biomarkers were measured at time of enrollment, prior to study drug administration, and at 72 hours. The primary endpoint in the trial was the change in IL-6 level from time zero to 72 hours between groups.

The secondary endpoint was to determine whether the administration of statin drugs attenuated disease severity and improved time to clinical resolution of symptoms in patients with confirmed influenza over the first 10 days after enrollment. Our main endpoint in this aim was the trend over time for resolution of the clinical illness based on a daily composite score of major influenza symptoms. Additional secondary clinical outcomes included hospital and intensive care unit (ICU) lengths of stay measured in days, rates of progression to vasopressor-dependent shock in each group, and in-hospital mortality.

### Biomarker Analysis

Influenza patient plasma samples were analyzed for multiple vascular endothelial and inflammatory markers (IL-6, tumor necrosis factor-alpha [TNF-α], vascular endothelial growth factor [VEGF], IL-2, IL-10 and vascular adhesion molecule-1 [VCAM-1]) using customized Meso Scale Discovery Human Multiplex Panel (Meso Scale Diagnostics, LLC, Rockville, MD). All samples were measured in duplicate with the interassay coefficients of variability ranging from 2.2–5.8%. We reported the VCAM-1 in log-transformed micrograms per milliliter (mL) and the rest of the markers in log-transformed picograms/mL.

### Statistical Analysis

Sample size calculation for the trial was based on a pilot clinical trial in which we randomized 18 patients with septic shock to 40 mg simvastatin—eight patients receiving atorvastin vs 10 receiving placebo daily. At the 72-hour time point, we found a change in IL-6 levels of (−964.3 ± 1501.2) in the statin group and (−471.9 ± 620.0) in the placebo group. Based on these numbers, we calculated 87 patients in each group to detect a change in biomarker levels between the statin and placebo groups at alpha 0.05 and 80% power. The trial was stopped at the end of the study funding period prior to achieving target enrollments.

We planned a *modified intention to treat* analysis for this trial defined by subjects who were consented, enrolled, randomized, and received at least one dose of the study drug. Prior to unblinding, as we had a number of patients who were lost to follow-up with only enrollment data available, we elected to perform a *per protocol* analysis for all patients who had 72-hour biomarker data available and had received all doses of study drug up to that point. We performed an additional post-hoc analysis of patients who presented within 48 hours of symptom onset to determine whether any effect of atorvastatin might be observed earlier in the clinical course, similar to antiviral therapy. Also prior to unblinding, we censored the biomarker results of one patient as only enrollment values and a second lab draw at 150 hours were available. The blood sampling occurred over two days after the last scheduled dose of study medication, and any imputed values may not reflect the effect of the drug.

Descriptive: We summarized the data using means with standard deviation or medians with interquartile ranges (IQR) for continuous variables and proportions (or frequencies) for categorical variables. Wilcoxon rank-sum tests were used to evaluate for the difference between groups for continuous variables and the chi-square or Fisher exact test for categorical variables, as appropriate.

#### Primary Endpoint

We compared biomarker levels between the two groups at each time point using a Wilcoxon rank-sum test. The distribution of these markers was assessed and transformed as necessary (eg, log transformation). Mean difference with a 95% confidence interval was calculated for the primary outcome, IL-6, at the 72-hour time point.

Hypothesis testing: We tested for group differences in biomarkers over time using a linear mixed-effects model to account for the correlation from within-subject measurements, testing several covariance structures. We chose the covariance structure based on the model with the lowest log likelihood value. We included an interaction between time and group, which was the primary outcome. A *P*-value <0.05 was considered significant.

#### Secondary Endpoints

We used Wilcoxon rank-sum tests to describe the differences in clinical symptoms over time between treatment groups. Both overall symptom resolution and resolution of individual components were described. Similar to the primary endpoint, we tested for group differences in reported symptoms over time using a linear mixed-effects model to account for the correlation from within subject measurements, including an interaction factor between time and group. For time to 50% symptom resolution, we performed survival analysis and used log-rank tests. For the secondary outcomes of hospital and ICU length of stay, we compared the groups using the Wilcoxon rank-sum test. We performed statistical analyses using Stata software, v14.2 (StataCorp, LLC, College Station, TX).

#### Role of the Funding Source

The funder of the study had no role in the study design, data collection, data analysis, data interpretation, or writing of the report. The corresponding author had full access to all the data in the study and had final responsibility for the decision to submit for publication.

## RESULTS

**Figure 1:**
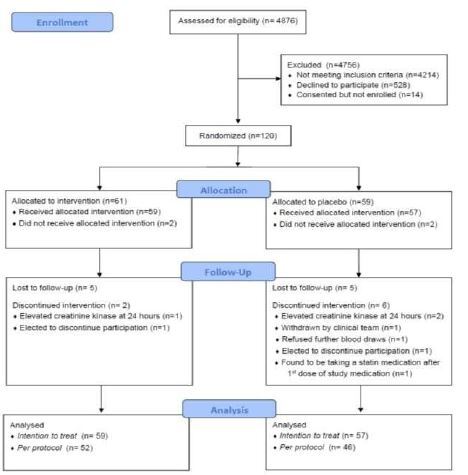
Screening and enrollment for clinical trial on the effect of atorvastatin vs placebo in patients with acute influenza infection.

We screened 4,876 patients presenting with influenza-like illness for the trial. Reasons for exclusion are presented in Figure 1. The most common exclusion was current statin use. We enrolled 120 patients between December 27, 2013–April 24, 2018. Two patients in each group discontinued participation prior to receiving the study drug. Fifty-nine patients were randomized to the atorvastatin group and 57 to the placebo group and received at least one dose of study drug. Treatment groups were similar in terms of baseline characteristics (Table 1). Initial clinical parameters between the groups were also comparable with regard to influenza immunization status, initial influenza severity of illness score, duration of symptoms, and medications administered during the ED stay (all *P* > 0.05). Rates of admission to the ICU, hospital ward, and ED observation unit were also similar between groups (*P* = 0.083), with the majority of patients (62.5%) being discharged from the ED.Ten patients were lost to follow up after the initial encounter. An additional eight patients withdrew from the study, two in the atorvastatin group and six in the placebo group, leaving 98 patients who achieved the primary endpoint in the trial, 52 in the atorvastatin group and 46 in the placebo group (Figure 1). This population is represented in the *per protocol* analysis group. There were no differences observed between the groups in safety lab testing at baseline, 24- or 72-hour time points. One patient in the atorvastatin group and two patients in the placebo group were found to have a creatinine kinase elevation above the specified safety lab parameters at the 24-hour time point and were discontinued from the trial.

**Figure 2:**
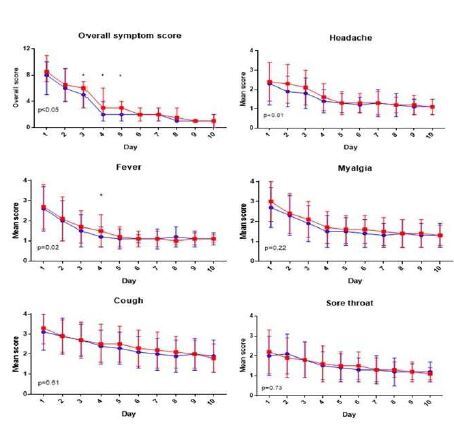
Biomarker mixed model analysis in a study of the effect of atorvastatin vs. placebo in acute influenza. Atorvastatin group in blue, placebo group in red. *IL*, interleukin; *TNF*, tumor necrosis factor; *VCAM*, vascular adhesion molecule; *VEGF*, vascular endothelial growth factor.

The primary endpoint in the trial was the change in IL-6 from enrollment to 72 hours. Mixed-model analysis of biomarker data did not detect any significant differences between the atorvastatin and placebo groups with regard to IL-6 levels (*P*=0.468) or any of the other biomarkers tested (Figure 2). This was also true when the analysis was restricted to the *per protocol* group (all *P* > 0.05). The mean difference for log-transformed IL-6 at the 72-hour time point was not significantly different between groups (−0.24, 95% CI −0.88–0.40).

**Figure 3:**
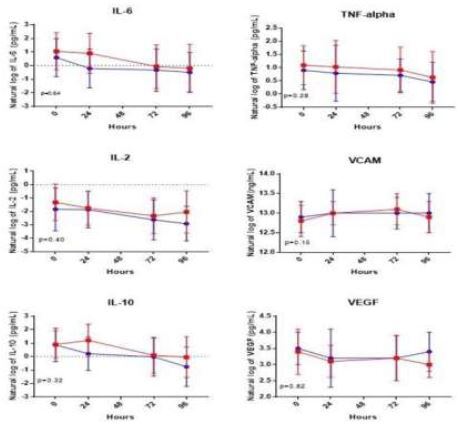
Influenza symptom severity mixed model analysis in this study examining the effect of atorvastatin in acute influenza. Atorvastatin group in blue, placebo group in red. *P*-values reflect the model over 10 days, asterisks denote days with significant differences between groups on individual days.

We observed a significant difference in symptoms severity, favoring faster resolution of symptoms in the atorvastatin group (*P*=0.049). This result was largely driven by the fever (*P*=0.013) and headache (*P*=0.010) components of the score. The result persisted in the *per protocol* analysis; *P*=.047 overall, *P*=.017 for fever and *P*=.003 for headache (Figure 3).

The effect on the overall symptom score reporting post study drug initiation was most pronounced on day 3, 4 and 5 of treatment (all *P*< 0.05). On day 3 of treatment, there were 21 (41%) patients in the atorvastatin group who reported return to normal activities compared to 12 (24%) in the placebo group, but this difference did not reach statistical significance (*P*=0.076). For the overall cohort, we did not detect any difference between groups with regard to reported time to 50% reduction in symptom scores. There were also no differences observed between groups with regard to ICU and hospital length of stay and no patients in the trial died or developed vasopressor-dependent shock (all *P*> 0.05).

**Figure 4:**
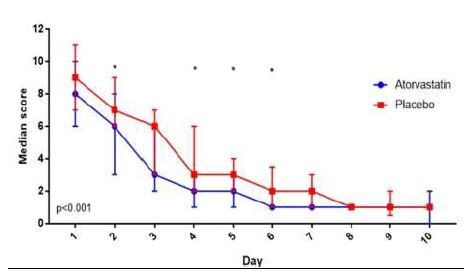
Influenza symptom severity score by treatment, patients with symptom duration ≤48 hours. In a study of the effect of atorvastatin vs placebo in acute influenza, this figure represents the self-reported influenza symptom severity scores in patients presenting within 48 hours of symptom onset.

When we restricted our analysis to patients who presented within two days of symptom onset (63 for *intention to treat*, 54 for *per protocol*), we observed minimal change in the biomarker analysis but found a significant difference between the groups for the symptom severity outcome (Figure 4). This was true for the overall score (*P* < 0.001) as well as the fever, sore throat, and headache components of the score in both the *intention to treat* and *per protocol* analyses. When examining median time to 50% resolution of symptoms in this subset of patients, we found that patients who received atorvastatin reported median resolution in 3 (IQR 2, 4) days vs 4 (IQR 3, 4) days in the placebo group, but this difference was not significant (*P*=0.36).

## DISCUSSION

We did not find significant differences between the atorvastatin and placebo groups with regard to inflammatory biomarkers over time, our primary outcome. However, atorvastatin appeared to be safe for patients with acute influenza infection and, most importantly, hastened resolution of influenza symptoms with the most significant effect of atorvastatin therapy observed in patients who presented within 48 hours of symptom onset.

### Biomarker Outcome

While much of the previous work assessing the effect of statins in infection has been observational or in animal models, there are several previous interventional studies in humans. These studies have generally found mixed results with respect to the effect of statin therapy on inflammatory markers and used lower doses of statin as compared to the present trial.^26^–^28^ We based our hypothesis about the contribution of inflammatory cytokines on poor outcomes in influenza infection on the existing literature, which substantiated these associations. However, given that we did not identify any differences between groups with regard to any of the inflammatory markers tested yet did appreciate improved clinical outcomes, we have to consider other mechanisms by which statins exerted their clinical benefit in our trial. There is a body of evidence that suggests that IL-6 is critical in the transition from innate to adaptive immunity in the response to influenza infection, albeit in animal models.^29^

Mechanistically, it is possible that the improved clinical outcomes observed resulted from one or a combination of the multiple other anti-inflammatory and immunomodulatory effects of statins not captured in our biomarker analysis. Sapey et al recently published the results of a pilot clinical trial on ICU patients with pneumonia. They randomized 62 patients to simvastatin 80 mg vs placebo and examined neutrophil function on day 4 of treatment.^30^ They found improved neutrophil function and lower Sequential Organ Failure Assessment scores in the simvastatin group but did not identify any significant differences in levels of inflammatory biomarkers including IL-6 and TNF-α. Alternatively, statins may have their greatest effect in a specific genotype of disease as one secondary analysis demonstrated in patients with acute respiratory distress syndrome (ARDS).^31^ The HARP-2 trial demonstrated no overall mortality benefit between the statin and placebo groups, but patients with the “hyperinflammatory” sub-phenotype who received simvastatin had significant improvement in 28-day survival compared to those who received placebo.

### Clinical Outcomes

With regard to our secondary clinical outcome, we found consistent and positive results for patients in the atorvastatin group. Notably, our atorvastatin group felt better as compared to the placebo group after two days of study drug, and this effect persisted for an additional two days compared to placebo. Additionally, we found that this clinical improvement was most pronounced in the subset of patients who had fewer than 48 hours of influenza symptoms. However, we did not detect a significant difference in median time to 50% resolution of symptoms in the subset of patients who presented early, highlighting the disparate results in these analytic approaches (survival analysis for time to 50% resolution compared to Wilcoxon rank-sum analysis for the differences in median severity score by day).

Several observational studies have also demonstrated that earlier initiation of neuraminidase inhibitors result in improved outcomes in influenza.^32^,^33^ In contrast, Beigel and colleagues conducted a randomized clinical trial administering oseltamivir alone or oseltamivir, amantadine, and ribavirin in combination. They observed a decrease in viral shedding on day 3, but this did not translate to improved clinical outcomes including duration of symptoms.^34^ We studied atorvastatin as a possible adjunctive therapy in acute influenza treatment and are strongly encouraged by the findings that patients felt better, faster on atorvastatin as compared to placebo.

There was a difference between groups with regard to return to normal activities that did not reach statistical significance, and there may be a variety of reasons for this including our sample size and precision of the assessment (days vs hours). Nonetheless, it follows that this could potentially translate into fewer missed work days, either for the employees themselves or as caregivers for a sick household member.^35^ We know that in the United States alone, the economic burden of seasonal influenza ranges from $11.2–87.1 billion annually when taking into account outpatient visits, hospitalizations, missed work days, and premature mortality.^36^,^37^ Given the potential public health and economic impact of fewer “sick” days with influenza, for this reason alone, atorvastatin therapy in acute influenza may be worthy of further study beyond the current trial.

Subsequent to the completion of this trial, severe acute respiratory syndrome coronavirus-2 emerged causing the COVID-19 pandemic and the associated deaths of millions of people worldwide. This has been a reminder of the potential impact of respiratory viruses on both individual and global health and the importance of finding additional therapeutic options for the treatment of respiratory viruses. A number of observational studies have found a correlation between statin use and clinical outcomes in COVID-19 patients and possibly provide additional support for the potential benefit of statins as an anti-inflammatory agent with pleiotropic effects in respiratory viral disease.^38^–^40^

### Safety

Our findings of safety mirror most trials of statin therapy in acute infection that have not found any significant safety concerns and adds data for patients with acute influenza infection who received atorvastatin, the majority (63%) of whom also received concurrent oseltamivir therapy. We are aware of a meta-analysis of six clinical trials using statin therapy in acute lung injury and ARDS. Although the authors found no benefits in any of the clinical outcomes, one trial did demonstrate potential harm with fewer days free of renal failure and fewer days free of hepatic failure in the treatment group as opposed to placebo.^41^ In this trial, ICU patients diagnosed with ARDS were randomized to receive a loading dose of rosuvastatin 40 mg followed by 20 mg daily.^42^ This dose is comparable to the 40 mg atorvastatin used in the present trial. While we did not observe any similar adverse effects, our trial had a small number of patients admitted to the ICU (7%); therefore, we were unable to draw any conclusions about safety in critically ill patients with acute influenza.

## LIMITATIONS

The trial did not reach target enrollments within the funding period as we were unable to add enrollment sites given the constraints of rapid screening laboratory tests and both storing and dispensing study drug to a largely outpatient population. However, our power calculations were based on highly conservative pilot data with wide standard deviations. We did have several participants in each group who were lost to follow-up after enrollment, but these were evenly distributed between the groups. This trial did not distinguish between patients with influenza A or influenza B and, therefore, we are not able to comment on potential differences between those groups. Our population was also fairly low acuity with two-thirds of them being discharged following their ED visit. Thus, our findings might not be generalizable to all populations. Because we did not have a rigorous program of monitoring study drug administration outside the hospital we cannot be certain that all study participants adhered to the scheduled dosing. However, we have no reason to believe that rates of compliance would be low given the willingness of participants to enroll in such a trial and return to the hospital for lab testing on day 3 or that there would be any difference between groups given the lack of any serious side effects reported.

## CONCLUSION

In this study we did not find any difference over time in pro-inflammatory biomarkers between patients with acute influenza treated with atorvastatin vs placebo. We did find a clinical benefit for patients in the atorvastatin group, which was most pronounced for patients early in their clinical course. Given that we and others have demonstrated the safety of statin medications, further study may be warranted to explore both the mechanisms and potential benefits from statin therapy in acute influenza infection.

## Figures and Tables

**Table 1:** Patient characteristics for influenza patients receiving atorvastatin vs placebo.

Variable n (%) unless otherwise designated	All Patients (N=116)	Atorvastatin (n=59)	Placebo (n=57)
Age (median, IQR)	37 (25, 54)	34 (23, 51)	43 (29, 58)
Sex			
Male	44 (38)	26 (44)	18 (32)
Female	72 (62)	33 (56)	39 (68)
Race			
White	74 (64)	34 (59)	40 (70)
Black	35 (30)	20 (34)	15 (26)
Other race	6 (5)	4 (7)	2 (4)
Social History			
Alcohol abuse	2 (2)	2 (3)	0 (0)
Tobacco use	7 (6)	6 (10)	1 (2)
Past Medical History			
None reported	45 (39)	24 (41)	21 (37)
Asthma	22 (19)	9 (15)	13 (23)
COPD	10 (9)	6 (10)	4 (7)
Diabetes	6 (5)	2 (3)	4 (7)
Cancer	6 (5)	3 (5)	3 (5)
Obesity	6 (5)	2 (3)	4 (7)
Hypertension	25 (22)	14 (24)	11 (19)
Hyperlipidemia	8 (7)	4 (7)	4 (7)
Liver disease	2 (2)	2 (3)	0 (0)
Renal disease	3 (3)	0 (0)	3 (5)
Stroke	2 (2)	1 (2)	1 (2)
Immunocompromised	7 (6)	2 (3)	5 (9)
Body Mass Index (median, IQR)	28.1 (23.8, 33.5)	26.2 (23.4, 33.9)	28.4 (26.4, 33.4)
Current influenza immunization			
Yes	44 (38)	21 (36)	23 (40)
No	71 (61)	38 (64)	33 (58)
Unknown	1 (1)	0 (0)	1 (2)
Influenza characteristics (median, IQR)			
Duration of symptoms in hours	48 (24, 72)	48 (45, 78.5)	48 (24, 72)
Symptom score at enrollment	8 (6, 10)	8 (6, 10)	8 (6, 11)
Disposition from ED			
ICU admission	8 (7)	4 (7)	4 (7)
Ward admission	24 (21)	7 (12)	17 (30)
ED observation	10 (9)	7 (12)	3 (5)
Discharged from ED	74 (64)	41 (69)	33 (58)
Medications administered in ED			
Antiviral	69 (59)	37 (63)	32 (56)
Antibiotics	26 (22)	12 (20)	14 (25)
Steroids	15 (13)	8 (12)	7(12)

*IQR*, interquartile range; *COPD*, chronic obstructive pulmonary disease; *ED*, emergency department; *ICU*, intensive care unit.
